# Supplementary material for: Physical Activity in 6.5-Year-Old Children Born Extremely Preterm
Source: J Clin Med. 2020 Oct 4;9(10):3206. doi: 10.3390/jcm9103206 (PMC7600509; doi:10.3390/jcm9103206)
Supplement: Supplementary file 1 [file jcm-09-03206-s001.pdf]

Percentage of time in 10 levels of PA, comparison index vs controls, stratified on sex.

	males			females		
	index mean (95% CI)	controls mean (95% CI)	p	index mean (95% CI)	controls mean (95% CI)	p
In cut point 1	62.7 (59.7, 65.6)	57.2 (54.7, 59.7)	**0.011**	57.5 (54.8, 60.2)	59.8 (57.1, 62.4)	0.263
In cut point 2	27.4 (25.5, 29.4)	29.0 (27.4, 30.6)	0.255	31.1 (29.4, 32.8)	29.8 (28.2, 31.5)	0.316
In cut point 3	7.0 (6.0, 8.0)	9.2 (8.3, 10.0)	**0.002**	8.3 (7.4, 9.3)	7.5 (6.6, 8.4)	0.251
In cut point 4	1.9 (1.4, 2.5)	3.0 (2.5, 3.5)	**0.007**	2.0 (1.6, 2.3)	1.9 (1.6, 2.2)	0.819
In cut point 5	0.7 (0.4, 1.0)	1.1 (0.8, 1.3)	0.068	0.7 (0.5, 0.9)	0.6 (0.5, 0.8)	0.765
In cut point 6	0.2 (0.1, 0.4)	0.4 (0.3, 0.5)	0.114	0.2, (0.1, 0.3)	0.2, (0.1, 0.3)	0.629
In cut point 7	0.0 (-0.0, 0.1)	0.1 (0.1, 0.2)	**0.036**[#]	0.1 (0.0, 0.2)	0.0 (0.0, 0.1)	0.443
In cut point 8	0.0 (-0.0, 0.0)	0.0 (0.0, 0.0)	**0.023**	0.0 (-0.0, 0.1)	0.0 (-0.0, 0.1)	0.820
In cut point 9	0.0 (-0.0, 0.0)	0.0 (0.0, 0.0)	0.144	0.0 (-0.0, 0.0)	0.0 (-0.0, 0.0)	0.711
In cut point 10	0.0 (-0.0, 0.0)	0.0 (-0.0, 0.0)	0.853	0.0 (-0.0, 0.0)	0.0 (0.0, 0.0)	0.083

PA- Physical Activity, index - children born extremely preterm, control - children born at term. All differences from cut-point 4 and above were additionally tested for significance with the Mann-Whitney U-test, since not normally distributed. [#]not sinificant in Mann-whitney U-test.
